# Ultrasonic Transducers Shaped in Archimedean and Fibonacci Spiral: A Comparison

**DOI:** 10.3390/s20102800

**Published:** 2020-05-14

**Authors:** Antonino S. Fiorillo, Salvatore Andrea Pullano, Maria Giovanna Bianco, Costantino Davide Critello

**Affiliations:** BATS Lab, Department of Health Sciences, University “Magna Graecia” of Catanzaro, 88100 Catanzaro, Italy; nino@unicz.it (A.S.F.); mg.bianco@unicz.it (M.G.B.); critello@unicz.it (C.D.C.)

**Keywords:** Archimedean spiral, ferroelectric polymer, Fibonacci spiral, piezoelectricity, cochlea, ultrasonic transducers bio sonar

## Abstract

We developed and investigated a particular geometry of transducers, emulating the shape of bats’ cochlea, to transmit and receive ultrasounds in the air. Their design involved the use of polyvinylidene fluoride (PVDF) as a piezoelectric material, thanks to its excellent conformability and flexibility. This material offers the primary requirements for sensing devices in applications such as sonar system or energy harvesting technology. The piezo film was folded according to both the Archimedean and Fibonacci spirals, and their performances were investigated in the frequency range from 20 kHz up to more than 80 kHz. The finite element analysis (FEA) of the proposed transducers highlighted the presence of multiple resonance vibrations, proved by the experimental measurements of the equivalent electric impedance and frequency response. Far-field radiation patterns demonstrated, horizontally and vertically, omnidirectional properties both as transmitters and receivers. All was enough to establish the best validity of the spiral shaped transducers for applications based on the bio sonar principle.

## 1. Introduction

Distance ranging evaluations using sonar systems are performed, in air or underwater, in a myriad of applications, in biomedical, civil, and military areas [1,2,3]. The use of low-frequency ultrasounds (US) for sonar tasks was first inspired by nature. In fact, certain species of mammals, such as bats and dolphins, have developed a built-in, target-locating mechanism, known as echolocation [4,5]. 

Previous investigations were directed towards reproducing US at certain fixed frequencies for specific applications; also because the most dominant technologies allowed the manufacture of piezoelectric transducers only in the frequency range of 40–50 kHz. Direct piezoelectric effect involves the conversion from mechanical into electrical energy. The opposite transduction applies to the converse piezoelectric effect [6]. With the advent of ferroelectric polymers [7], new opportunities were offered to scientists in the field of acoustic sensors. Referring to sonar systems, the supremacy of polyvinylidene fluoride (PVDF) has been widely demonstrated for the design and manufacturing of short-range US transducers in the air from 30 kHz up to 100 kHz, which is the same frequency range used by bats [8,9,10,11,12]. The only limitation pertained to the working frequency, which was fixed around a central value, failing to cover the whole band of interest. The first attempt to mimic the spectrum of bats’ bio sonar dates to 1996 [13]. However, that device, equipped with a mechanical system for the changing of its resonant frequency, was too heavy and bulky to be mounted on small mobile robots or wearable systems. Since then, the PVDF film was arranged according to various geometries [14,15,16], widening the operating frequency band to no more than 11 kHz. 

For mobile robots, what is of primary importance is not only detecting the target’s position, but also mapping the surrounding space. One of the main limitations of US transducers is the inability to see in all directions, which results in poor radial resolution. Such a limitation depends on the radiation characteristics of piezo polymer devices, which, in turn, depend on the spatial configuration of the film itself. For instance, transducers with a semi conical geometry have a radiation beam in the horizontal plane with angles from 50° to 80° at 26 and 35 kHz, respectively [14]. Chen at al. truncated the semi conical geometry, obtaining significant improvements of the beam’s aperture on both the horizontal (omnidirectionality) and vertical (140°) planes, but this was only at the resonant condition of the transducer (33 kHz) [15]. Commercial cylindrical PVDF transducers, which resonate at 40 and 80 kHz only, have vertical beam directivities of 80° and 50°, respectively. Omnidirectionality, instead, still concerns only the horizontal plane.

Recently, a bat echolocating auditory apparatus suggested the design of an ultrasonic transducer based on light and flexible PVDF, folded according to a logarithmic spiral [17,18]. This geometry allowed a significant increase in the operating frequency range, showing omnidirectional characteristics as transmitters and receivers, both in the horizontal and vertical planes. Following the enthusiasm of this discovery, we developed and characterized piezo polymer transducers with different spiral geometries, which have their own spatial conformation. In addition to the logarithmic spiral, those known as Archimedean and Fibonacci spirals have also been used to represent the coiled shape of the mammalian cochlea [19,20], and this was our fundamental reason for adopting these spiral-like geometries to design a PVDF transducer for irradiating and receiving low-frequency US. The acoustic and electrical characterization of Archimedean- and Fibonacci-shaped transducers was investigated in the low-frequency range from 20 kHz up to 80 kHz. The finite element analysis of the proposed transducers highlighted the presence of multiple resonance vibrations, proved by experimental measurements of the equivalent electric impedance and frequency response. Far-field radiation patterns demonstrated, horizontally and vertically, omnidirectional properties both as transmitters and receivers. The article’s novelty was to establish the influence of the cochlear shape on the characteristics of US sensors. Finally, we recognized that the logarithmic spiral [17,18] was the best shape for designing US transducers providing omnidirectionality in the whole space and over the whole large band.

## 2. Bio Inspiration and Conception

Among the species of echolocating bats, mustached bats exploit US calls falling in the range from about 20 kHz up to 120 kHz [21]. The anatomy of the bat auditory system is similar to that of other mammals, including human beings [22,23]. Piezo polymer transducers we proposed in this article mimicked bats’ basilar membrane in the spiral shape, and their working principle was based on the inverse proportionality between the resonance frequency and the bending radius [8,9,10,11] (as in standard piezo ceramics with curved geometry [24]). Particularly, spiral shaped transducers can be considered as the summation of arc portions of different lengths, each of which increases from the center to the periphery along the spiral, corresponding to a continuous decreasing of the vibration frequency [18].

**Figure 1:**
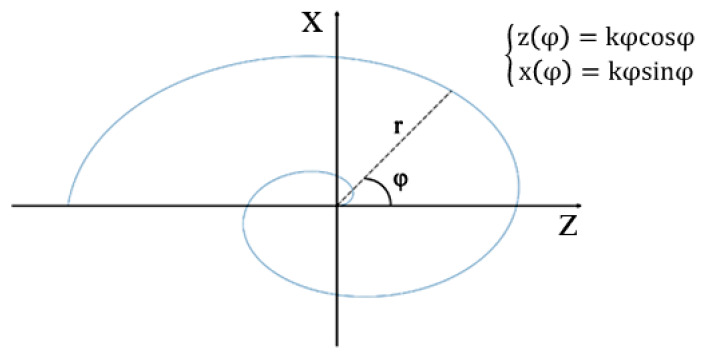
Archimedean spiral (not to scale) function with the set of parametric equations.

Spiral geometries have been observed in nature, from small living systems such as snails, to big phenomena such as hurricanes, and even in the case of the crystal growth of nanostructured zeolites [25,26,27,28]. In the macro world, the mammalian cochlea is a very common natural evidence of spiral coiling, and the Archimedean and Fibonacci spirals are the nonlinear curves often used for its description [19,20]. The Archimedean spiral is a curve generated by a point moving uniformly along the radius vector, which rotates around its origin point (pole). The two-dimensional polar equation of an Archimedean spiral is:

r(φ) = k φ,(1)

where r is the radius vector, k is the arbitrary constant, and φ is the angle between the polar radius and the x-axis. Unlike the logarithmic spiral, the Archimedean one maintains the same distance between consecutive turnings, and this is where the other name, “equidistant spiral”, comes from. Figure 1 shows the Archimedean spiral and its parametric description. In this work, the independent parameter was defined in the range from 0 up to 3π (i.e., three half-turns), assuming that the constant k equals 0.06. According to the clamped film theory, the choice of three half-turns (0–3π) imposes the theoretical lower frequency limit at around 20 kHz (radius vector of 24 mm), and the upper frequency limit at around 80 kHz (innermost portions of the spiral).

**Figure 2:**
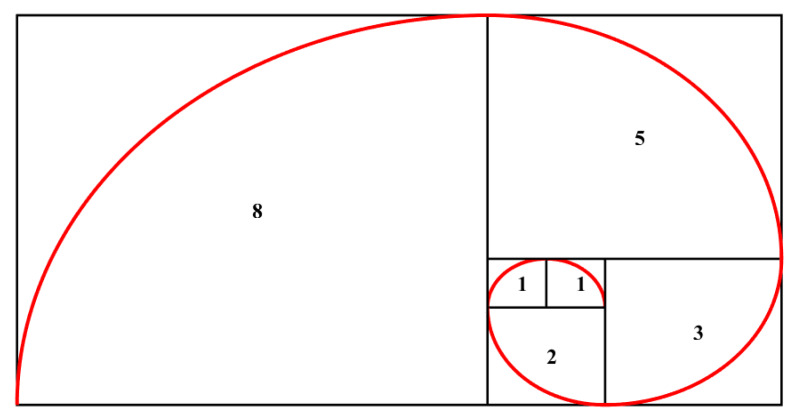
Fibonacci spiral (not to scale). Each square has side lengths referring to the Fibonacci sequence.

**Figure 3:**
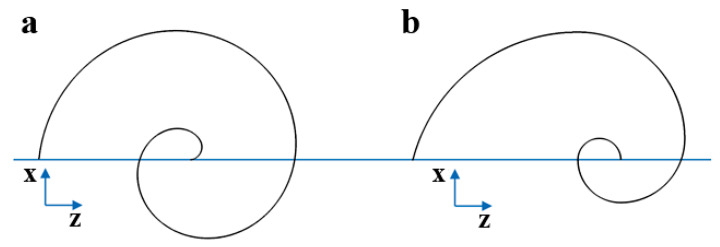
Comparison between the two-dimensional (**a**) Archimedean and (**b**) Fibonacci spirals according to the same scale.

The Fibonacci spiral comes from the famous sequence in which any number is the sum of the two numbers preceding it. The curve can be built geometrically using squares with side lengths representing the Fibonacci numbers. Quarter-circle arcs are then drawn to connect the opposing corners of the squares, resulting in a spiral shaped curve. Figure 2 illustrates the Fibonacci spiral using the first six numbers of the sequence (excluding the number zero). In Figure 3 is reported a comparison between the two geometries using the same scale.

## 3. Materials and Methods

**Figure 4:**
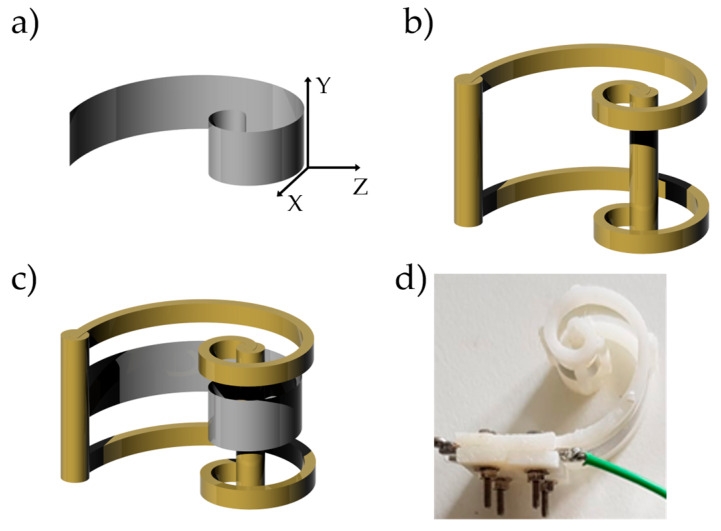
Fabrication of ultrasonic transducer which includes (**a**) a 3D printed Acrilonitrile butadiene stirene (ABS) support, and (**b**) a sheet of PVDF whose piezoelectric direction is properly oriented. The latter is finally inserted inside the slits of the support before being mechanically and electrically connected to the (**c**) support and the electronic interface. Representative fabrication of one of the (**d**) proposed spiral geometries.

The fabrication process of the US devices started with the design of the Archimedean-like and Fibonacci-like plastic components (12 mm × 16 mm), using the Stratasys uPrint SE 3D printer with fused deposition modeling (FDM) technology (Figure 4a), acting as the support to impose the desired geometry to the PVDF film. The US transducers, poled in the Z-direction and folded around the Y-axis (Figure 4b), have been designed such that they can vibrate in radial mode by blocking only the two extremities (Figure 4c,d). When, due to electric or mechanical stimulus, the PVDF film vibrates, such a radial mode determines US irradiation or the registration of a voltage signal, respectively.

**Figure 5:**
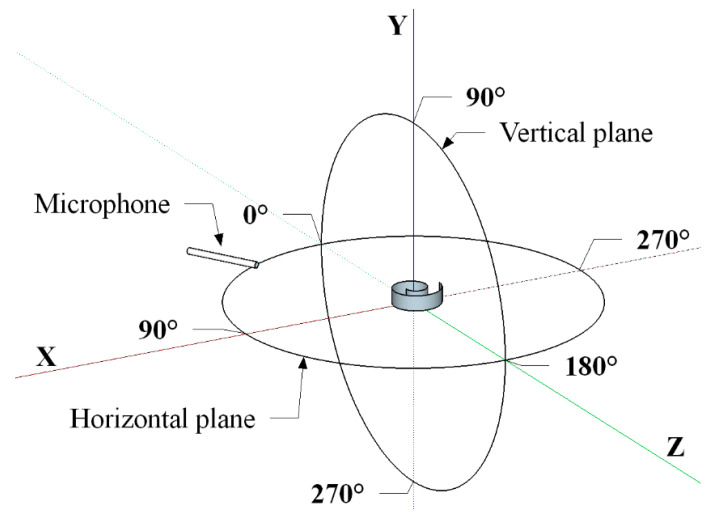
Experimental setup (not to scale) to measure the transmitted radiation pattern in both the horizontal and vertical plane. The distance separating the microphone, which can be manually moved with 15° steps, and the ultrasounds (US) transmitter, was equal to 0.3 m.

**Figure 6:**
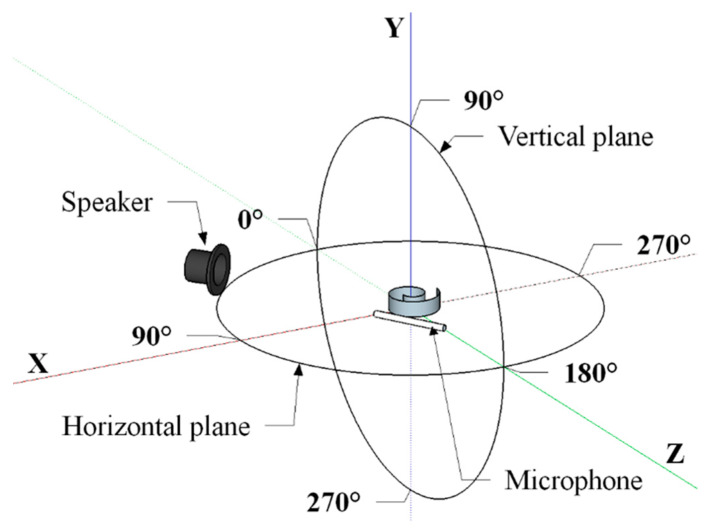
Experimental setup (not to scale) to measure the received radiation pattern in both the horizontal and vertical plane. The distance separating the speaker, which can be manually moved with 15° steps, and the US receiver, next to the microphone, was equal to 0.3 m.

Figure 5 shows the experimental setup used for directivity measurements. As for the acquisition of the transmitted US signals, face-to-face configuration (0.3 m distance) included the free-field calibrated microphone type 4939 (Brüel & Kjær, Nærum, Denmark), which could be manually moved at different angles (15° steps), and the centrally located spiral-like transducer (Figure 5). The Nexus 2690 (Brüel & Kjær, Nærum, Denmark) was used as the charge amplifier for conditioning the US response. Sound pressure levels (SPLs) were evaluated driving the PVDF transducer with a 5-cycle, 150-V_pp_ sinusoidal burst using a reference pressure of 20 μPa (0 dB scale). As for the received signals (Figure 6), the spiral shaped US transducer was excited by using a super tweeter driver (Murata ESTD01), being characterized by a broad spectrum (15–100 kHz). In order to irradiate a constant pressure level (1 Pa) in the frequency range from 20 kHz up to 80 kHz, the free-field calibrated microphone (Brüel & Kjær type 4939) was placed next to the US receiver. The receiver sensitivity (S) was evaluated driving the piezo speaker with a 5-cycle, 150-V_pp_ sinusoidal burst. The output response of the Archimedean- and Fibonacci-shaped transducers was then conditioned by a custom low-noise pre-amplifier (60 dB voltage gain).

A precision LCR meter (Keysight E4980A/AL, Santa Rosa, CA, USA), coupled with a 16089B test fixture, was used to evaluate the impedance of the Archimedean- and Fibonacci-shaped transducers over a wide range of frequencies (20 to 80 kHz), using a test signal level with an amplitude of 2 V_rms_.

Frequency response was investigated using the pulse-echo technique. The following components were employed: a function generator (Tektronix AFG 3102, Taipei City, Taiwan) providing a 150 V, a 100 μs rectangular pulse, a device under test—DUT (i.e., US transducer), a detector (microphone Brüel & Kjær type 4939, Nærum, Danish), a broadband amplifier (Brüel & Kjær Nexus 2690, Nærum, Danish), and an oscilloscope (Tektronix DPO3054). A fast Fourier transform (FFT) of the signal, acquired at 0.3 m distance, was finally performed for the evaluation of the spectral response.

Two-dimensional finite element modeling (FEM) allowed us to study the resonance behaviors of the US sensor, using COMSOL Multiphysics ver. 5.4 (COSMOL Group, Burlington, Massachusetts, USA). The model considered a thin film (25 µm thick) of PVDF material folded according to the spiral (i.e., Archimedean and Fibonacci) shape. The domain (without the surface metallization) was a 2D, linear elastic, isotropic piezoelectric material, referring to the XZ plane. The piezo material was characterized by a density of 1770 kg/m^3^ and a relative electric permittivity equal to 12. The β-phase PVDF (with C_2v_ or 2 mm symmetry) allowed us to consider five piezoelectric coefficients and nine elastic compliance coefficients. The characteristic constitutive equations, including the mechanical and piezoelectric parameters, are reported below:

(2) $$\left|\begin{array}{c} S_{1}\\ S_{2}\\ S_{3}\\ S_{4}\\ S_{5}\\ S_{6}\\ D_{1}\\ D_{2}\\ D_{3} \end{array}\right|=\left|\begin{array}{ccccccccc} s_{11}^{E} & s_{12}^{E} & s_{13}^{E} & 0 & 0 & 0 & 0 & 0 & d_{31}\\ s_{12}^{E} & s_{22}^{E} & s_{23}^{E} & 0 & 0 & 0 & 0 & 0 & d_{32}\\ s_{13}^{E} & s_{23}^{E} & s_{33}^{E} & 0 & 0 & 0 & 0 & 0 & d_{33}\\ 0 & 0 & 0 & s_{44}^{E} & 0 & 0 & 0 & d_{24} & 0\\ 0 & 0 & 0 & 0 & s_{55}^{E} & 0 & d_{15} & 0 & 0\\ 0 & 0 & 0 & 0 & 0 & s_{66}^{E} & 0 & 0 & 0\\ 0 & 0 & 0 & 0 & d_{15} & 0 & \varepsilon_{11}^{T} & 0 & 0\\ 0 & 0 & 0 & d_{24} & 0 & 0 & 0 & \varepsilon_{22}^{T} & 0\\ d_{31} & d_{32} & d_{33} & 0 & 0 & 0 & 0 & 0 & \varepsilon_{33}^{T} \end{array}\right|.\left|\begin{array}{c} T_{1}\\ T_{2}\\ T_{3}\\ T_{4}\\ T_{5}\\ T_{6}\\ E_{1}\\ E_{2}\\ E_{3} \end{array}\right|=\left|\begin{array}{ccccccccc} 3.65 & -1.92 & -2.09 & 0 & 0 & 0 & 0 & 0 & 21\\ -1.92 & 4.24 & -1.92 & 0 & 0 & 0 & 0 & 0 & 2.3\\ -2.09 & -1.92 & 4.72 & 0 & 0 & 0 & 0 & 0 & -26\\ 0 & 0 & 0 & 4.24 & 0 & 0 & 0 & -27 & 0\\ 0 & 0 & 0 & 0 & 4.24 & 0 & -23 & 0 & 0\\ 0 & 0 & 0 & 0 & 0 & 9.5 & 0 & 0 & 0\\ 0 & 0 & 0 & 0 & -23 & 0 & 12 & 0 & 0\\ 0 & 0 & 0 & -27 & 0 & 0 & 0 & 12 & 0\\ 21 & 2.3 & -26 & 0 & 0 & 0 & 0 & 0 & 12 \end{array}\right|.\left|\begin{array}{c} T_{1}\\ T_{2}\\ T_{3}\\ T_{4}\\ T_{5}\\ T_{6}\\ E_{1}\\ E_{2}\\ E_{3} \end{array}\right|$$

where [T_i_] is the stress vector, [D_i_] is the electric displacement vector, [E_i_] is the electric field vector, [d_ij_] is the piezoelectric constant matrix (values expressed as 10^−10^ C/N), [ε_ij_^T^] is the relative electric permittivity matrix, and [s_ij_^T^] is the elastic compliance (values expressed as 10^−10^ Pa^−1^) [18,29,30]. The analysis was conducted on the two different domains using the structural (COMSOL Solid Mechanics interface) and electrical (COMSOL Electrostatics interface) modules, and properties were assigned. All the domains were considered free to move, except for the clamped edges. The material was considered linear, elastic, and isotropic, with piezoelectric characteristics, according to the data reported in Equation (2). Both domains were meshed by free tetrahedral elements and solved in the frequency domain (eigenfrequency analysis), evaluating the absolute value of the displacement along the domain.

## 4. Results and Discussions

PVDF is a semi crystalline fluoropolymer, characterized by excellent piezoelectric and pyroelectric properties, chemical resistance, and mechanical strength [31]. Recently, a logarithmic spiral-like US transducer, which was inspired by the shape of the mammalian cochlea, demonstrated significant improvements in terms of bandwidth and omnidirectional directivity [16,17]. The shape of the cochlea has been also associated with the Archimedean and Fibonacci spirals [19,20], and this prompted us to design transducers folded according to these geometries for in-air transmission and reception at low-frequency (20–80 kHz) US.

**Figure 7:**
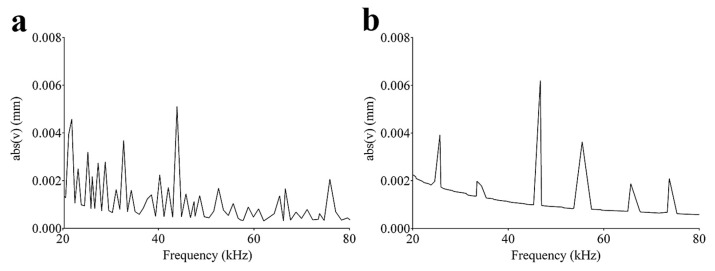
Displacement (absolute value) of the (**a**) Archimedean and (**b**) Fibonacci spirals, evaluated in the range of 20–80 kHz. Each peak reflects a natural mode of vibration.

Finite element analysis was performed to investigate the eigenfrequencies of the models, i.e., the natural frequencies of the vibration modes. In Figure 7a,b, the absolute value of the displacement in the frequency range from 20 kHz up to 80 kHz highlighted multiple resonance frequencies of the Archimedean and Fibonacci spirals. Previous investigations pointed out that a PVDF transducer, folded according to the logarithmic spiral, can effectively be modeled as a summation of contiguous semi cylindrical transducers vibrating at discrete frequencies, albeit each mode of vibration interested the whole geometry [18]. The Archimedean spiral exhibited greater displacement, particularly at lower frequencies, than that observed for the Fibonacci geometry (Figure 7a,b). The latter emphasized a few vibrating peaks since the whole geometry has been obtained (as indeed it has) from a limited number of circle arcs (six in our case), obeying more strictly to the clamped PVDF theory. Moreover, both spirals evidenced a displacement characterized by a mean value which decreased as a function of frequency.

**Figure 8:**
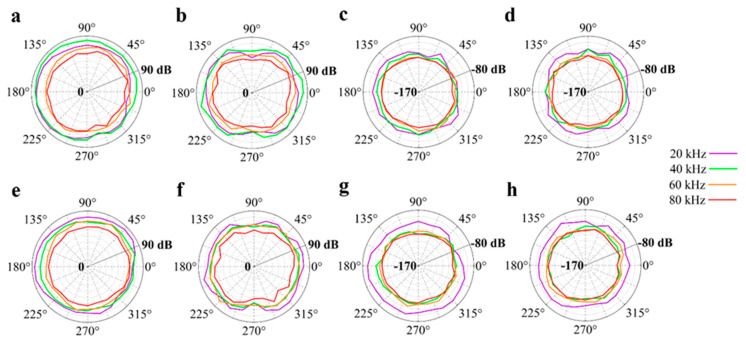
Experimental polar plots of the radiation patterns for the (**a**–**d**) Archimedean- and (**e**–**h**) Fibonacci-shaped transducers used as (**a,b,e,f**) transmitters and (**c,d,g,h**) receivers in both the (**a,c,e,g**) horizontal and (**b,d,f,h**) vertical planes.

First experimental measurements concerned the radiative characteristics of the PVDF film used as an actuator and sensor. Figure 8 shows the radiation patterns of the Archimedean and Fibonacci spirals in the horizontal and vertical planes, considering both the transmission (SPL) and the reception (S) of US signals. Generally, the dB values decreased as a function of increasing frequency. This could depend on greater US attenuation and/or, according to the clamped PVDF theory, the vibration of arc portions on the spiral with smaller bending radii [18]. Table 1 (Archimedean spiral) and Table 2 (Fibonacci spiral) illustrate the minimum and maximum values of transmitted and received dB values at 20, 40, 60, and 80 kHz in the azimuthal and axial planes for both the spiral shaped geometries. The Fibonacci geometry had better performances at low frequency (20 kHz), especially as a receiver. Comparing the SPL and S values related to the Archimedean, Fibonacci, and logarithmic [17,18] spiral shaped transducers, it was clear that the logarithmic spiral resulted as the best electromechanical device to emit and detect ultrasonic signals.

**Figure 9:**
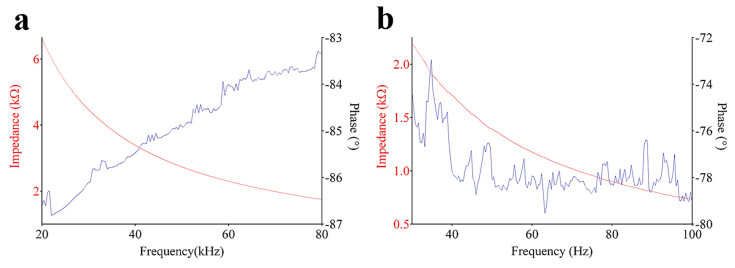
Experimental electrical impedance (module and phase) in the frequency range from (**a**) 20kHZ up to 80kHZ, (**b**) 40kHZ up to 100kHZ, for the Archimedean and Fibonacci spiral-shaped transducers.

The piezoelectric transducer can be electrically modeled according to the common Butterworth–Van Dyke (BVD) equivalent circuit, consisting of a static branch in parallel with a dynamic branch. The first includes a capacitor in parallel with a resistor, both affected by the frequency; the second involves a (resonant) series of resistor, inductor, and capacitor. In order to make insensitive the static branch from the frequency in the range of 1–150 kHz, a modified BVD model has been proposed, involving the addition of a parallel connection with an RC series [32]. Recently, a further modification on the BVD equivalent electrical circuit has been adopted for modeling PVDF transducers folded according to the logarithmic shape, including multiple series branches, to reproduce the multiple resonant modes of vibration [17]. In this article, the electrical impedance has been experimentally evaluated for the Archimedean- and Fibonacci-shaped transducers. Figure 9a,b shows the module and phase curves of the impedance as a function of frequency, from 20 kHz up to 80 kHz. As for the case of the logarithmic spiral geometry [17], the phase curves of both transducers highlighted several peaks, meaning that the film exhibited multiple vibration modes along its structure.

**Figure 10:**
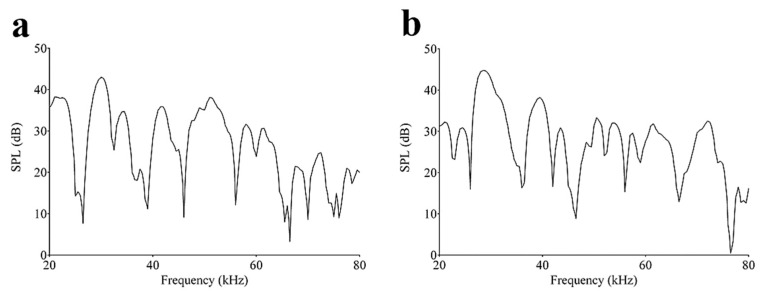
Frequency response of the (**a**) Archimedean and (**b**) Fibonacci spiral-like transducers excited by 150 V, 100 μs rectangular pulse.

The frequency response of a piezoelectric US transducer usually has a band-pass shape, which determines the range of frequencies over which the transducer itself can operate with the highest efficiency in terms of energy conversion. As far as our transducers are concerned, the frequency band of interest, from 20 to 80 kHz, was not shaped in the usual way. Instead, multiple resonant frequencies came out using a rectangular pulse as an excitation signal (Figure 10). The prevailing peak was around 45 kHz, in accordance with the displacement of the spiral geometry obtained from FEM analysis. No center frequency was, therefore, observed in the spectrum; so, not a single value of Q factor could be determined for the proposed transducers, but many Q factors can be associated to each resonant frequency. A similar situation was found for the logarithmic spiral geometry, even if its spectrum appeared broader and less selective [17]. What is important to emphasize about Figure 8 is the presence of multiple resonances that would represent multiple semi cylindrical resonators, each of which is ruled by the classical theory of curved, clamped film.

## 5. Conclusions

Bio sonar refers to the innate echolocating skill of some mammals, such as bats and dolphins, to use low-frequency US to obtain information about the surrounding environment for a matter of survival. In some species of bats, this information is created by processing broadband US signals. In this paper, US transducers have been designed, being inspired by the shape of the mammalian cochlea. PVDF thin films were folded according to two peculiar geometries: those of the Archimedean and Fibonacci spirals. Simulated and experimental data confirmed that the transducers with a spiral geometry resonate at multiple frequencies in a broad frequency range, emitting and receiving US signals in all directions, both in the azimuthal and axial planes. These characteristics have not been found in the related prior art, excluding the logarithmic spiral-shaped transducer with a very similar geometry. The proposed transducers might be, therefore, easily integrated in those systems such as the assistive devices for visually impaired individuals, to accomplish a better detection of targeted obstacles, improving the level of satisfaction and safety during daily activities.

## Figures and Tables

**Table 1:** Maximum and minimum of dB values obtained from the directivity measurements for the Archimedean spiral-like transducer.

	Transmission				Reception			
	Horizontal Plane		Vertical Plane		Horizontal Plane		Vertical Plane	
Frequency (kHz)	Max (dB)	Min (dB)	Max (dB)	Min (dB)	Max (dB)	Min (dB)	Max (dB)	Min (dB)
20	84	66	79	57	−95	−112	−95	−109
40	85	69	85	62	−101	−115	−101	−113
60	74	64	72	58	−104	−117	−105	−115
80	68	56	66	52	−107	−116	−105	−117

**Table 2:** Maximum and minimum of dB values obtained from the directivity measurements for the Fibonacci spiral-like transducer.

	Transmission				Reception			
	Horizontal Plane		Vertical Plane		Horizontal Plane		Vertical Plane	
Frequency (kHz)	Max (dB)	Min (dB)	Max (dB)	Min (dB)	Max (dB)	Min (dB)	Max (dB)	Min (dB)
20	86	68	84	58	−87	−109	−92	−105
40	79	68	75	59	−102	−118	−105	−118
60	78	64	79	62	−103	−114	−102	−114
80	69	60	67	52	−109	−119	−110	−119
